# Characteristics of Leadership Competency in Nurse Managers: A Scoping Review

**DOI:** 10.1155/2024/5594154

**Published:** 2024-07-16

**Authors:** Silvia Perez-Gonzalez, Pilar Marques-Sanchez, Arrate Pinto-Carral, Alberto Gonzalez-Garcia, Cristina Liebana-Presa, Carmen Benavides

**Affiliations:** ^1^ Faculty of Health Sciences Nursing and Physiotherapy Department Universidad de León, León 24007, Spain; ^2^ Faculty of Health Sciences Nursing and Physiotherapy Department SALBIS Research Group Campus de Ponferrada Universidad de León, León 24401, Spain; ^3^ Department of Electric Systems and Automatics Engineering SALBIS Research Group Universidad de León, León 24007, Spain

## Abstract

**Aim:**

Identify the characteristics of leadership competency for the nurse manager and describe the most cited leadership styles in the literature.

**Background:**

Leadership is a fundamental competency for nurse managers, as it plays an important role in the healthcare environment to achieve Sustainable Development Goals and promote people-centered organizations. Therefore, understanding the characteristics of leadership and the leadership style to be employed is important.

**Methods:**

A scoping review was conducted from January 2009 to January 2024 using the design of González Garcia et al., the Arksey and O'Malley framework, and databases including Web of Science, Scopus, and PubMed. Articles reporting on the characteristics of leadership for nurse managers were reviewed. The authors performed the review based on a search syntax, inclusion and exclusion criteria, and the data extraction process.

**Results:**

Sixty-two studies were included in the final review. The review identified 38 characteristics related to leadership competency, among which we highlighted caring for nurses as individuals, being a visionary, knowledgeable, a change agent, and a communicator. This review highlights the prevalence of transformational leadership, which constitutes 69.57% of the leadership styles cited, and underscores its pivotal role in improving the work environment, effectiveness in nursing care, conflict management, team commitment, and adaptability to change within healthcare settings.

**Conclusions:**

The most commonly cited characteristics of leadership include caring for the team, effective communication, and a vision for change. Transformational, people-centered, and motivational leadership is the most appropriate style. *Implications for Nursing Management.* The characterization of leadership competency will allow the development of training adapted to the current requirements for nursing leaders. This training could be developed in simulation and virtual reality environments. It also allows for a deeper understanding of how leadership competency affects teams and their functioning.

## 1. Introduction

Leaders in healthcare organizations must inspire coordinated actions and foster a social movement to achieve the Sustainable Development Goals (SDGs), which require the development of people-centered ethical behavior [1, 2]. This leadership style can be defined as one that focuses on achieving the highest levels of motivation of people through the development of four components: idealized influence, inspirational motivation, intellectual stimulation, and individual consideration [3]. They should be responsible for positive changes in social and health systems throughout the world, taking into account not only the interests of the organization but also the interests of the society in which it operates [4]. The SDGs refer to the global challenges facing countries and their populations [5].

Nurses are associated with the SDGs, as their work involves concern for the health impact on patients and populations [6]. It is important to note that nurses, from a crucial position, address social determinants of health, understanding the link between public health conditions and their impact on the population [7]. In this sense, the nurse should exercise a participatory and collaborative leadership style within work groups and society [8]. Furthermore, nurses' leadership focused on the SDGs would have clear benefits related to improving global health, progress toward gender equality, and strengthening economies [9]. Similarly, the World Health Organization (WHO) highlights the relationship between nursing leadership and its influence on education (SDG 4), gender relations (SDG 5), and work and economic growth (SDG 8) [10]. Benton et al. [11] also noted that the 17 SDGs have a significant impact on people's health, underscoring the crucial role of nursing leadership in coordinating actions to achieve these goals.

Furthermore, the nurse managers play a relevant role in the success of healthcare institutions in the management of governance, quality, and sustainability, as well as in terms of health objectives and the SDGs [12]. In addition, nurse managers establish strategies that guide nurses in their professional actions, generate environments for nursing practice, and support organizational development, influencing public health policies and positioning nurses in an ever-changing healthcare environment [13]. Thus, developing competencies to effectively manage healthcare organizations becomes crucial, especially when these roles serve as a vital link to clinical practice [14].

In this sense, the competency model for the nurse manager developed by González Garcia [15] provides a core of eight competencies that nurses must develop in managerial roles in healthcare organizations [16]. These core competencies described by González Garcia [15] include leadership.

The leadership competency of the nurse manager could be defined as the ability to influence other professionals to achieve common objectives and a shared vision [17, 18]. This competency is especially important in healthcare organizations because it is responsible for generating professional commitment, reducing conflicts in workgroups, and creating relationships between team members [19]. Furthermore, research has found that the leadership style of the nurse manager is a key determinant of the quality of care provided by nurses [20, 21]. Research also identifies the transformational leadership style as the most relevant style in the healthcare setting [18, 22] due to its ability to achieve commitment, reduce stress, prevent burnout, and achieve better health outcomes and higher quality nursing care [23, 24].

The relationship between the SDGs, leadership, economic, and sustainability policies with respect to the need to provide quality healthcare is the starting point for the present research [25–27]. However, the review of the literature indicates that it is necessary to deepen the knowledge of leadership competence and its characteristics in the healthcare setting [28–30].

### 1.1. Aim

The objectives of this scoping review were: (1) to identify and describe the characteristics of leadership competency for the nurse manager and (2) to identify the most common leadership style in the literature.

## 2. Materials and Methods

### 2.1. Design

A scoping review synthesizes knowledge, explanations, and interpretations from both qualitative and quantitative research to address the research question. A scoping review is useful for exploring emerging evidence, identifying knowledge gaps, and providing rigorous and transparent methods for identifying and mapping available evidence [31]. This methodology enables the systematic and meaningful extraction of data [32]. According to Tricco et al. [33], this research adhered to the checklist proposed by PRISMA-ScR.

In addition, this scoping review followed the methodology conducted by Gonzalez-Garcia et al. [34], as this article belongs to the line of research focused on the development of competencies for nurse managers. The same keywords were used to refer to the nurse manager, as well as the same approach to competencies and characteristics. Likewise, the same article selection and quality criteria were used. Regarding data extraction and coding systems, the same tools and strategies adapted to the purpose of the competency that is the focus of this part of the research were used.

### 2.2. Search Methods

The scoping review was conducted according to Arksey and O'Malley [31], which involved: Identifying the research question, identifying relevant studies, choosing studies, identifying data, collecting, summarizing, and reporting results.

The research questions were as follows:

1. What are the characteristics of leadership competency for the nurse manager?
2. What are the characteristics of leadership competency that are most often cited in the literature?
3. What is the most cited leadership style in the literature?

Relevant articles published between 2009 and 2024 were searched in electronic databases, including Web of Science, Scopus, and PubMed. The search terms included references to nurse managers and leadership competency (Table 1).

The inclusion criteria for the articles in this scoping review included quantitative, qualitative, and mixed methods studies, theses and dissertations, and reviews. Articles published between January 1, 2009, and January 30, 2024, in English or Spanish, were included to guarantee the synthesis of updated knowledge, considering that the role of nurse managers and leadership has evolved in recent years. The exclusion criteria included conference abstracts, editorials, and discussion papers, as well as articles with no data on the leadership characteristics of the nurse manager.

We limited our search to the articles that resulted from our search equations and did not include gray literature. Additionally, we did not conduct a snowballing search.

### 2.3. Quality Appraisal

An evaluation of the quality of the included articles was performed. Since developed and validated tools for assessing the different methodologies of the included publications are lacking, the development of a specific tool to serve this purpose was necessary. To this end, parts of the method presented by Hölbl et al. [35] were used and modified as appropriate (Table 2). The initial quality assessment of the articles was performed by SPG and was independently reviewed by reviewers 2–6 (PMS, AP, CB, AGG, and CLP). Although some studies had medium scores, the authors' intention was not to remove studies but to assess the overall quality of the existing knowledge base. No articles were excluded according to the quality assessment process.

### 2.4. Data Extraction

Data extraction was performed using specially designed forms. The following data were extracted from the studies: type of study, sample size, participant characteristics, countries, leadership types, and leadership characteristics. Data analysis was performed using Microsoft Excel.

The authors met regularly to resolve any disagreements about whether the articles met the inclusion criteria. Each of the selected full-text articles was read thoroughly several times by the authors to capture all relevant information and ensure that nothing important was missed. The data set for the paper was constructed by extracting findings that were relevant to the research questions.

Studies, characteristics, and leadership styles were coded using the following process to correctly identify them:

  Articles: Coded with the letter “A” followed by three digits, beginning with A001 for the first article.
  Characteristics: Coded with the letter “C” followed by three digits, beginning with C001 for the first characteristic.
  Leadership style: These were encoded with the letter “L” followed by three digits, beginning with the code “L001.”

For example, the characteristic encoded as “A003 C007” can be identified as characteristic 7 belonging to Article 3. Furthermore, the leadership style encoded as “A003 L003” can be identified as leadership style 3, as described in Article 3.

Using this methodology, we initially cataloged the characteristics of leadership competency described in the articles and assigned a unique code to each. Subsequently, these characteristics were analyzed and grouped if they were identical, and their frequency of repetition was tallied. Any disagreements were resolved by a fourth reviewer (CB).

In this review, the “leadership characteristic” is defined as a description that distinctly recognizes a leadership trait.

## 3. Results

**Figure 1:**
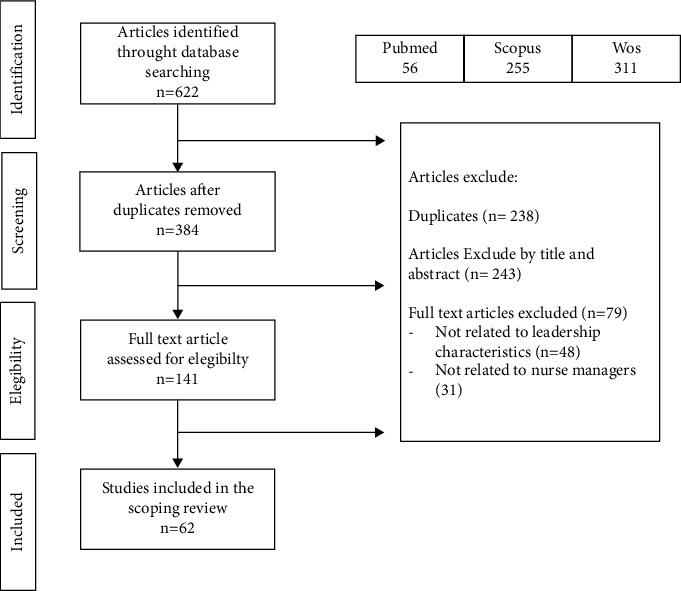
Flowchart of the study.

The selection process is shown in Figure 1. A total of 622 articles were identified from electronic databases and imported into Mendeley for screening. After screening the titles and abstracts, 141 studies remained for full-text review. Finally, 62 studies were included in this scoping review.

### 3.1. Bibliographic Overview

Sixty-two articles were included in the current scoping review.  Table 3 presents the characteristics of the included studies. Of the 62 studies, most were conducted in the USA (54.8%), Finland (7.54%), Iran and China (5.66% each), Australia and Jordan (3.77% each), and 1.88% came from the UK, Saudi Arabia, Singapore, Greece, South Africa, Italy, Japan, Brazil, Lithuania, and Sweden. As a result, 339 characteristics were identified in the 62 articles analyzed. Following the identification of the characteristics, their meanings were analyzed, and those referring to the same leadership characteristics were grouped together. This analysis resulted in 38 leadership characteristics (Table 4).

### 3.2. Quality Assessment

The highest possible score was 10, while the lowest was 0. The overall average score was 6.70. The average scores for the individual items were as follows: *Q*1 = 1.34 ± 0.51, *Q*2 = 1.49 ± 0.60, *Q*3 = 1.05 ± 0.43, *Q*4 = 1.59 ± 0.64, and *Q*5 = 1.21 ± 0.55.

### 3.3. Most Cited Characteristics

Regarding the five most cited leadership characteristics in the literature, each with a frequency greater than five, they were “Caring for nurses as individuals,” “Being visionary” (creating and clearly communicating a personal vision to guide change and enable others to achieve their purpose and take action), “Knowledgeable,” “Change agent,” and “Being a communicator” (Table 4). This requires a combination of personality traits and communication skills.

### 3.4. Leadership Styles

Furthermore, the leadership styles from which the extracted characteristics originated were analyzed, including a study of their frequencies, resulting in 22 leadership styles, as shown in Table 5. Given the way leadership was referred to in the various articles, it was pertinent to analyze and classify them based on how they were defined. From this analysis, four leadership styles emerged (Table 6). A significant finding was that 69.57% of the articles referred to transformational leadership, as seen in Table 6.

Consequently, the identified leadership characteristics were classified into the four defining dimensions of transformational leadership (Table 7). The most cited characteristics in each of the four dimensions were: in the intellectual stimulation dimension, the most cited characteristic was “knowledgeable” (14.49%); in the individualized consideration dimension, the most cited characteristic was “Caring for nurses as individuals” (32.34%); “Empowerment” (25.42%) was the most cited in the idealized influence dimension; and “Being visionary” (28.57%) was the most cited in the inspirational motivation dimension.

Subsequently, an analysis was conducted on the influence of leadership on key aspects of nursing team management, such as job satisfaction, effectiveness in patient care, conflict management, team commitment, and adaptability to change in the nursing environment, as shown in Table 8. The results indicate that transformational and relational leadership achieve the highest levels in all areas of analysis, demonstrating a significant impact on job satisfaction, effectiveness in patient care, conflict management, team commitment, and adaptability to change.

## 4. Discussion

The objectives of this scoping review were to identify the characteristics of leadership competency for nurse managers and to identify and describe the leadership styles most commonly referenced in the literature. The strengths of our methodology include rigorously defined inclusion criteria, quality assessment steps, and the use of a nurse manager competency model validated by a broad panel of experts [96].

We identified 339 characteristics of leadership competency, although this number was reduced to 38 after each characteristic was analyzed individually and grouped according to its meaning. In the scientific literature, only one study classifies the characteristics of competencies but differs in how it classifies the characteristics of leadership. For example, the “ethics” characteristic is not included within leadership but rather as a separate dimension [97]. On the other hand, the analyzed works consistently specify certain specific attributes in the leadership of nurse managers, often not referring directly to leadership characteristics or discussing leadership in a general way without delving into the competence itself [58, 98].

When comparing some of the characteristics identified in this review with those of other studies that discuss competencies, we observed similarities with our findings. Li and Wivatvanit [66] also prioritized characteristics related to the personal domain in their research, such as self-confidence, serving as a role model for staff, and being innovative and creative. The importance of innovation becomes even more crucial in constantly changing environments, such as healthcare [66].

Our research reveals leadership characteristics related to communication, such as “Being a communicator” and “Listening,” within the Individualized Consideration dimension. In this line of thought, Hopkinson et al. [56] also emphasize communication as a key characteristic in the leadership of nurse managers. Anderson et al. [39] demonstrated how the ability of nurse leaders contributes to creating collaborative environments, reducing costs associated with healthcare, and improving patient safety.

A particularly relevant characteristic that emerges from our research is “knowledgeable,” as it is the fourth most frequent and most frequently cited within the intellectual stimulation dimension. The importance of this characteristic was emphasized by Lehtonen et al. [64], who prioritized the training of nurse managers in exercising leadership within work teams.

The literature reviewed in this research suggests that nursing leaders must focus on emotional intelligence as one of the fundamental pillars of the leadership of nursing teams, in addition to ensuring care efficiency and patient safety. Bikmoradi et al. [43] echoed this opinion by stating that emotional intelligence is the most relevant aspect that leaders must develop to achieve excellent performance in their work teams.

Furthermore, our research revealed that transformational leadership is predominant in the context of nursing management, with 69.57% of the findings focusing on transformational leadership. This result aligns with those reported by Ferreira et al. [99], Sammut and Scicluna [100], and Duggar [101], who identify transformational leadership as responsible for the best outcomes for nurses and patients, making it suitable for the development of nurse manager roles. Boamah et al. [102] added that transformational leadership exercised by nurse managers is key to nursing team satisfaction, work environment, achieved outcomes, and staff retention.

Regarding the impact of transformational leadership, our research shows that this leadership style is the most relevant for the work environment, the effectiveness of nursing care, conflict management, team commitment, and adaptation to change. Sahan and Terzioglu [103] and Jankelova and Joniakova [104] point out how transformational leadership has a high impact on all organizational outcomes, especially on job satisfaction of nurses. Lappalainen et al. [63] evidenced a positive correlation between transformational leadership and patient safety.

Additionally, Alise [105], in her thesis studies, identifies transformational leadership as responsible for the outcomes of conflict management. Likewise, Bagga et al. [106] show how transformational leadership is significantly related to change management and organizational culture, aligning with Lewans [107], who points out transformational leadership as key in the implementation of changes in organizations.

## 5. Conclusions

This scoping review identified and described the characteristics of the leadership competency required for nurse managers, which is key to advancing health management toward the Sustainable Development Goals (SDGs). A total of 339 characteristics were identified in the 62 articles analyzed, which were synthesized into 38 characteristics and categorized into four distinct dimensions, providing a comprehensive structure for understanding the leadership of the nurse manager.

Five characteristics were identified as the most cited in the literature included in the review: caring for nurses as individuals, being a visionary, knowledgeable, agent of change, and being a communicator. The two most frequently cited characteristics were caring for nurses as individuals and being visionary. This underscores the need for nurse managers to adopt a visionary and person-centered leadership style.

The leadership style mentioned most frequently in this review was transformational, focusing on its impact on the work environment, the effectiveness of nursing care, conflict management, team commitment, and change management. Characterized by being person-centered, motivating, and inspiring, transformational leadership appears to be the most appropriate style for nurse managers, oriented toward the SDGs and changing environments of healthcare systems.

Emotional intelligence in the leadership of nurse managers has emerged as a fundamental pillar for improving the efficiency of the nursing team and patient care, in addition to favoring the achievement of health objectives, reducing conflict, and improving the work environment.

Future research should focus on developing various scenarios in which to train leadership competency, as well as evaluating the development of these competencies. Practical applications include integrating these identified characteristics into training programs for nursing managers to enhance leadership effectiveness and organizational outcomes.

## 6. Limitations

One limitation of this scoping review is the potential variability arising from the diverse contexts represented in the analyzed articles. Although the research team implemented measures to mitigate this challenge and comprehensively capture the knowledge base, some context-specific nuances may affect generalizability.

Moreover, despite employing multiple databases and search strategies in both English and Spanish, relevant studies published in other languages or gray literature sources may have been inadvertently excluded. This could introduce a potential bias toward the general representation of leadership characteristics and styles in nursing management. However, the research team believes that this risk is minimal given the extensive number of sources consulted in the previous phase of the research. We believe that the results presented bring us very close to knowledge saturation.

## 7. Implications for Nurse Managers

This research shows a series of characteristics relevant to the development of leadership competency that could be considered when developing training programs for nursing managers.

Undoubtedly, the identified characteristics constitute a body of knowledge necessary to develop the work of a nursing manager. Given the large number of characteristics and the difficulty involved in developing them, the characteristics identified as most cited represent the minimum characteristics that a nurse manager should develop. This body of knowledge leads to the operationalization of the competencies of person-centered, forward-looking leadership.

## Figures and Tables

**Table 1:** Search strategy.

Database 2009–2024	Search terms
WOS	TI = (leader∗) AND TI = (“nurs∗ manage∗” OR “nurse supervisor” OR “nurse program manager” OR “nurse unit manager” OR “chief nurse executive” OR “nurs∗ administrat∗” OR “director of nurs∗” OR “head nurs∗” OR “frontline manager” OR “nurs∗ director” OR “nurs∗ executiv∗”) AND TS = (“head nurs∗” OR “frontline manager” OR “nurs∗ director” OR “nurs∗ manag∗” OR “first line nurs∗ manage∗” OR “nurse supervisor” OR “nurse program manager” OR “nurse unit manager” OR “chief nurse executive” OR “nurs∗ administrat∗” OR “director of nurs∗” OR “nurs∗ executiv∗”) AND TS = (leader∗)
SCOPUS	TITLE-ABS-KEY(“nurs∗ manage∗” OR “nurse supervisor” OR “nurse program manager” OR “nurse unit manager” OR “chief nurse executive” OR “nurs∗ administrat∗” OR “director of nurs∗” OR “head nurs∗” OR “frontline manager” OR “nurs∗ director” OR “nurs∗ executiv∗”) AND TITLE-ABS-KEY(leader∗)
PUBMED	(leader∗) AND ((“nurse administrators”[Majr] OR “nursing, Supervisory”[Majr])

Source: own elaboration.

**Table 2:** Quality assessment tool adapted from Hölbl et al. [35].

Domain	Indicator (0–2)
Q1-Is the nurse manager leadership characteristics described?	No-moderately-yes
Q2-Are the research objectives clearly outlined?	No-moderately-yes
Q3-Are the main contributions well described to the nurse management leadership?	No-moderately-yes
Q4-How appropriate is the problem-solution fit?	No-moderately-yes
Q5-Are the proposed solutions feasible (scalable, economical, implementable)?	No-moderately-yes

**Table 3:** Description of the articles included in the review.

Author/year	Method	Description/context	Results	Limitations
(Abdelhafiz et al., 2016) [36]	Quantitative, descriptive, and comparative method	The aim is to explore how nurse leaders' leadership styles affect nurses' job satisfaction	The increased development of transformational leadership behaviors increases nurses' job satisfaction and thus contributes to higher nurse retention	Not described
(Albagawi, 2019) [37]	The multifactor management questionnaire was applied to evaluate supervisors' methods of management	Hail City Public Hospitals	The objective is to determine the management style of nurse managers and its consequences in Hail public hospitals	Not described
(Al-Hussami et al., 2017) [38]	Pre-experimental design	61 nurses participated, with a pretest and posttest group in Jordan	The objective is to establish a leadership orientation program that can advance nurses' knowledge while improving their leadership skills and quality of work to promote their readiness for change in healthcare organizations	Not described
(Anderson et al., 2010) [39]	Survey and focus groups	To assess aspects of leadership that help create a healthy work environment that supports the delivery of quality care	The authors discuss their findings and propose a theoretical model to explain the specific characteristics of nursing leadership that support job satisfaction and retention of nursing staff	Not described
(Batson and Yoder, 2009) [40]	Descriptive	Analyzes the influence of nurse manager coaching as a transformational leadership skill and describes an effective coaching process for staff	Although each employee is unique, generational differences that directly affect coaching have been identified	Not described
(Bellack and Dickow, 2019) [41]	Descriptive	The leadership literature is rich with research on characteristics and behaviors that cause leaders to derail	This research examines career derailment risks and explores opportunities to prevent or recover from leadership failure	Not described
(Bernard, 2014) [42]	Descriptive	The Centura Nurse Executive Residency Program is designed for nursing leadership development	Developing high-potential leaders is a critical factor for preparing a cadre of nurse executives	Not described
(Bikmoradi et al., 2018) [43]	Cross-sectional study using Bradberry's emotional intelligence and Metzcas and Bardon's leadership style questionnaires	370 nurse managers from hospitals of Hamadan University of Medical Sciences were surveyed	The emotional intelligence of nurse managers has a significant positive correlation with a people-oriented leadership style and, in contrast, no correlation with a task-oriented leadership style	The emotional intelligence and leadership style of the managers were measured from the managers' own perspectives
(Bish et al., 2015) [44]	Qualitative descriptive study	Semistructured in-depth interviews with rural nursing managers to identify factors influencing leadership	Recognizing factors that influence rural nurse leaders' approaches can help implement context-sensitive leadership development initiatives	Not described
(Cathcart et al., 2010) [45]	Narrative experiences on professional practice	Utilizing Benner's methodology of practice articulation, 32 nurse managers wrote and interpreted first-person narratives of their practice	Complex leadership challenges can be a significant source of experiential learning for individuals and groups	Not described
(Cheng et al., 2018) [46]	Qualitative interviews	Secondary qualitative analysis of 15 nurse manager interviews	Nurse manager leadership is crucial for the successful implementation of evidence-based nursing	There is a potential risk of overinterpretation in secondary data analysis studies
(Clement-O'Brien et al., 2011) [47]	Survey method	Directors of acute care hospitals participated in a survey to determine their capacity for innovation	Managers who completed more leadership courses had implemented significantly more types of innovations and had higher scores on the innovation scale	The selection of the state setting limits the generalizability of the findings
(Ebrahimzade et al., 2015) [23]	Cross-sectional study with questionnaire	207 hospital nurses completed a questionnaire assessing demographic characteristics, a measure of burnout, and the Multifactor Leadership Questionnaire (MLQ)	This study sheds light on the effective role of transformational leadership in improving nursing management and reducing burnout among nurses	Not described
(Echevarria et al., 2017) [48]	Predictive correlational design	148 nurse managers used the Genos Emotional Intelligence Inventory, the Multifactor Leadership Questionnaire, and a demographic questionnaire	The study's results underscore the need for continuing education programs in emotional intelligence, leadership development, and leadership assessment	It does not determine the participants' regions and practice environments
(Fennimore and Wolf, 2011) [49]	Descriptive study	The UPMC leadership development for nursing Middle managers program was designed within a conceptual framework. Twenty-five nurse managers participated in the pilot program	Effective nurse manager leadership is vital for successful hospital outcomes, leading to increased nurse retention, reduced turnover costs, and improved quality and financial outcomes for healthcare institutions	There was minimal opportunity to measure the longitudinal impact of the program
(Fischer et al., 2018) [50]	Systematic review on leadership and safety climate and expert panel	A group of 25 international experts in leadership and safety participated in a Delphi study with three rounds of Likert-scale surveys	There is a consensus on specific factors that play a significant role in an organization's safety climate. Generally, the leader's demonstration of commitment to safety is key to cultivating a patient safety culture	Not described
(Flury, 2017) [51]	Descriptive opinion	Nursing leaders in the USA must include social media in their communication strategy to lead healthcare transformation	Social networks, when used wisely, can be a valuable leadership communication tool for today's nurse leaders	Not described
(Gergely, 2018) [52]	Qualitative surveys and interviews	132 nurse leaders from AONE were electronically surveyed on influences on the delivery of culturally competent care delivery; interviews with six nurses	Cultural competence as a strategic priority can be improved by providing training and holding managers accountable	Not described
(Goh et al., 2018) [53]	Survey	Nurses in four inpatient wards of an acute hospital in Singapore completed various leadership and commitment questionnaires	RNs observed nurse leaders who showed transformational and transactional behaviors, with less laissez-faire behavior	Not described
(Grubaugh and Flynn, 2018) [54]	Secondary analysis	Data from 257 nurses across 50 medical-surgical units used for secondary analysis, originally collected from a 2012 study	The findings emphasize that the skilled leadership and conflict management of the nurse manager are crucial	Limited by the variables and measures of the original study
(Henriksen, 2016) [17]	Descriptive opinion	Framework for leadership transformation requires redefining the role of the nurse manager	Identifies six key attributes of a chief nursing officer and the effectiveness of an innovative learning approach	Not described
(Heuston and Wolf, 2011) [55]	Descriptive	Discussion on Kouzes and Posner's model and the five practices of exemplary leadership	Highlights the increasing value of leaders who can transform the workforce amid healthcare reforms	Not described
(Hopkinson et al., 2019) [56]	Literature review and focus groups	Constructs identified through literature search and refined via focus groups	Eight constructs emerged in regard to leadership development methods	Communication training was not covered
(Hughes, 2019) [57]	Oral history method	Examines Air Force nursing development through the experiences of a notable military nurse leader between 2004 and 2008	Provides information on the development and impact of air force nursing in a historical, social, and global context	Oral history centered around the experiences of a single nurse
(Kallas, 2014) [58]	Cross-sectional survey	Profile of an excellent nurse manager developed through responses from highly rated US nurse managers	The leadership attributes and competencies of an excellent nurse manager are outlined, in response to the nursing shortage	The tools necessary to identify these attributes are not present in the literature
(Kelly et al., 2014) [59]	Cross-sectional descriptive survey	Survey of 512 hospital managers to assess leadership characteristics	Formal training affects one component of transformational leadership (TL), helping leaders model behavior for employees	The path to leadership positions in nursing remains unclear
(Khan et al., 2018) [60]	Descriptive correlational design	Survey at 2016 Magnet Conference, including full-time nurses with 6+ months of experience	Moderate correlation between transformational leadership of nurse managers and structural empowerment of staff; less so for transactional leadership	The sample is convenience-based, primarily from magnet hospitals or those pursuing the designation
(Kodama et al., 2016) [61]	Cross-sectional study	Nurses assessed leadership styles of nurse managers and related factors of engagement	Intellectual stimulation from transformational leadership can increase staff retention by increasing affective engagement	The convenience nature of the sample limits generalizability
(Kvist et al., 2019) [62]	Cross-sectional survey	Nurses evaluated nurse leaders using the transformational leadership scale (TL)	Nurse managers' qualities were rated moderate by nurses	E-surveys tend to have low response rates
(Lappalainen et al., 2020) [63]	Electronic questionnaires	Finnish nurses responded to electronic questionnaires incorporating the transformational leadership scale (TLS) and the medication safety Scale (MSS)	There is a relationship between nurse managers' transformational leadership and medication safety	The response rate to the questionnaire was not specified
(Lehtonen et al., 2018) [64]	Electronic questionnaire	Nursing staff in Finland evaluated their manager using a leadership and management competencies scale in 2016	Nurses highly value the professional competence in nursing leadership and management. Greater appreciation requires managers to demonstrate education and competence	The results are limited to the specific hospital where the research was conducted
(Liukka et al., 2018) [65]	Semistructured interviews	Eleven nurse managers were interviewed about their actions after adverse events	Certain transformational leadership elements are significant in nurse managers' actions after adverse events	Participant feedback may have increased study validity
(Li and Wivatvanit, 2016) [66]	Delphi technique	Twenty experts participated, focusing on leadership competencies for first-line nurse managers	The consensus on competencies can guide leadership development programs in Shanghai, China	Not described
(Mackoff et al., 2013) [67]	Participatory action research	One-year study to develop “leadership laboratories” with nurse managers as participants and evaluators	Positive results in leadership skills from all laboratories	Not described
(Manning, 2017) [68]	Descriptive correlational design	Survey of 441 nurses in the USA using engagement and leadership questionnaires	Transactional and transformational leadership positively influenced engagement; passive leadership had a negative effect	Not described
(McKinney et al., 2016) [69]	Survey method	Surveys on leadership and intention to leave completed by 3,609 nursing directors	Complexity leadership approaches were correlated with better care outcomes	Not described
(Millet and Porche, 2017) [70]	Descriptive opinion	Discussion on leadership during natural disasters	The unique talents of nurses are crucial in health emergencies and disasters	Not described
(Morsiani et al., 2017) [71]	Two-phase mixed method	Initial phase using the Multifactor leadership questionnaire to associate leadership style with job satisfaction	Italian nurse managers must improve their transformational leadership skills	Not described
(Negussie and Demissie, 2013) [72]	Nonexperimental correlation design	Inclusion of full-time nonsupervisory nurses with more than a year of experience	Higher satisfaction with transformational leadership styles over transactional leadership styles	Not described
(Palweni et al., 2023) [73]	Qualitative exploratory and descriptive study	Explored nurse managers' perceptions of leadership styles and their impact on patient safety in an academic hospital	Identified common leadership styles among nurse managers and challenges to improve patient safety	Limited to one health sector; could benefit from more in-depth data
(Pereira et al., 2015) [74]	Quantitative study	Study with 15 nurses in hospital management in Brazil	Leadership is linked with communication and ethics; limited training opportunities postgraduation	Team strength is a barrier to leadership exercise
(Pishgooie et al., 2019) [75]	Cross-sectional and correlational study	Survey of 1,617 nurses in Iranian government hospitals	Transformational and transactional leadership styles can reduce nurse job stress	Not described
(Player and Burns, 2015) [76]	Descriptive opinion	Discussion on the universality of basic leadership skills	Leadership skills are essential, regardless of the career path; mentoring is a strong motivator	Not described
(Prado-Inzerillo et al., 2018) [77]	Quantitative descriptive study	Survey of 56 hospital chief nursing officers to describe leadership and participation	First study to add to the development of leadership of executive-level nurse leaders in Magnet hospitals	Not described
(Prezerakos, 2018) [78]	Review	Search in electronic databases (PubMed, Scopus, CINAHL) for articles published in English or Greek from 2000 to 2017	Emotional intelligence is a useful tool for nurse leaders and contributes significantly to effective healthcare management	Not described
(Qtait, 2023) [79]	Systematic review of quantitative studies	Study examining the impact of head nurses' leadership styles on nurse performance	Transformational and democratic leadership styles positively impact nurse performance	Not described
(Reyes et al., 2013) [80]	Qualitative study with in-depth interviews	Interviews with 7 directors of nursing about leadership challenges and characteristics during 2008-2010	Challenges include leadership dissonance and leading through ambiguity. Suggests supporting leaders with defined competencies	Not described
(Richey and Waite, 2019) [81]	Organization-wide employee engagement survey	Ann and Robert H. Lurie Children's Hospital/Chicago	The evaluation of data led to the development of the leadership engagement academy (LEA) for frontline nurse managers	Not described
(Saiki et al., 2023) [82]	Cross-sectional study	Assessing the Japanese version of the implementation leadership scale (ILS) among nurse managers and staff nurses	The Japanese ILS is a reliable and valid tool for evaluating leadership in the implementation of evidence-based practice	Not described
(Sandström et al., 2011) [83]	Systematic literature review	Review of leadership and its influence on the implementation process	The review included seven articles and identified three main areas of findings	Not described
(Sharpp et al., 2019) [84]	Interviews and focus groups	Nurse managers' perspectives on technology, communication influence and leadership in the US healthcare system	Nurse leaders need training to fully utilize technology for patient care and management	Participating nurses did not fully share their experiences
(Sherman et al., 2013) [85]	Action-research design for program development and evaluation	Develop and promote an innovative master's degree program in nursing administration for emerging young leaders	Emerging nurse leaders may be well positioned with the right skills to lead in the new era	Not described
(Smama'h et al., 2023) [86]	Descriptive correlational cross-sectional study	Examines the relationship between the leadership styles of nurse managers and the motivation and intentions of nurses to change in Jordan	Supportive leadership style scored highest. Positive correlation with nurses' motivation, but no significant correlation with turnover intention	Focus on private hospitals in Jordan, potential self-report bias, limited generalizability
(Spano-Szekely et al., 2016) [87]	Descriptive study to correlate emotional intelligence and transformational leadership practices of nurse managers	Convenience sample of nurse managers at the Magnet 2014/USA conference	Emotional intelligence was positively correlated with transformational leadership	Self-assessment nature of the study
(Swinton and Haverkamp, 2023) [88]	Quality improvement project	Evaluate the effectiveness of nurse leader laboratories in improving leadership competencies among nurse managers	Postintervention increases in emotional intelligence assessment scores and nurse manager skills inventory categories	Small sample size, focus on a specific health system
(Tau et al., 2018) [89]	Quantitative, descriptive, and correlational design	Two questionnaires were used: Wagnild and Young's resilience scale questionnaire and empowering leadership questionnaire	Relationship between nurse manager resilience and leader behavior; high resilience scores linked to greater leader empowerment	Limited to one health sector; individual discussions could provide more in-depth information
(Tyczkowski et al., 2015) [90]	Descriptive and exploratory study design	Study to determine the level and relationship between emotional intelligence and leadership style of nurse managers	Positive relationships found between emotional intelligence and transformational leadership outcomes	Not described
(Valiga, 2019) [91]	Descriptive	Article detailing characteristics of effective leaders and followers, emphasizing leadership outside of managerial positions	Various ways to identify, develop, and strengthen leadership skills	Not described
(Verschueren et al., 2013) [92]	Review on leadership styles	Literature review conducted from January 2000 to September 2011, across multiple databases	A trend suggesting the importance of trust between head nurses and subordinates for positive patient outcomes	Not described
(Walker et al., 2011) [93]	Narrative review	Critical evaluation of selected articles on the influence of leadership on organizational learning and development and undergraduate clinical education	Core leadership factors identified include transformational principles and the role of the nursing unit/CEO	Not described
(Yañez et al., 2016) [94]	Delphi technique	Consensus study among 67 staff members from various hospital services, divided into four groups	Consensus on behaviors displayed by leaders that foster trustworthiness	Not described
(Yoon et al., 2023) [95]	Cross-sectional descriptive online survey design	Study on the mediating effect of patient participation culture between ethical leadership and performance in Korean hospitals	Ethical leadership directly affects performance; patient participation culture partially mediates this relationship	Bias from the self-reported survey and focus on Korean hospitals, limiting generalizability

Source: own elaboration.

**Table 4:** Characteristics of leadership competency and frequencies.

Characteristics	Freq. abs	Freq. rel (%)
Care about nurses as individuals	22	6.49
To be visionary (create and clearly communicate a personal vision to guide change and enable others to achieve a purpose and move to action)	20	5.90
Knowledgeable	20	5.90
Change agent	19	5.60
Being a communicator (versatility—the ability to create a culture based on multiple modes of communication, situational judgment, change management, interpersonal relationships, and continuous feedback systems)	17	5.01
Effective conflict management (intergroup and intragroup conflicts)	16	4.72
Empowerment	15	4.42
Outcomes oriented	12	3.54
Innovation	11	3.24
Establish core values of the nursing team and develop the concept of teamwork	11	3.24
Share decision making	10	2.95
Coaching: Behaviour that educates team members and assists them to become self-reliant	10	2.95
Will and commitment	9	2.65
Promote strategies where staff can use ICT (information and communication technology) fluidly	9	2.65
Practice according to a certain standard for nursing administration practice-assess, plan, intervene, and evaluate	9	2.65
Ethics	8	2.36
Self-control	8	2.36
Inspire	8	2.36
Creativity	8	2.36
Empathy	7	2.06
Serve as a role model for staff	7	2.06
Enthusiasm	7	2.06
Provide autonomy	7	2.06
Understand healthcare economics knowledge, such as unit-cost analysis and cost-benefit analysis	7	2.06
Credibility	6	1.77
Accessible	6	1.77
Listening	6	1.77
Adaptive	6	1.77
Trusted	5	1.47
Emotional intelligence	5	1.47
Charismatic	5	1.47
Building positive relationships with your staff	5	1.47
Visibility	4	1.18
Cultural competence	3	0.88
Passion	3	0.88
Motivate	3	0.88
To be a critical thinker (acquire knowledge and practice using reflective learning cycle skills, such as planning, acting, observing, and reflecting)	3	0.88
Resilience (not only have the ability to survive in difficulty and adversity but are able to display behaviour that will enhance subordinates' ability to thrive)	2	0.59

Source: own elaboration.

**Table 5:** Overview of leadership styles: frequency and prevalence analysis.

Leadership style	Abs freq.	Rel freq. (%)
Transformational leadership	33	47.14
Transactional	8	11.43
Empowerment-based leadership	3	4.29
Innovative leadership	2	2.86
Situational leadership	2	2.86
Complexity leadership	2	2.86
e-leadership	1	1.43
Experiential leadership	1	1.43
Authentic	1	1.43
Situational	1	1.43
Strategic leadership	1	1.43
Reflective leadership	1	1.43
Ethical leadership	1	1.43
Competent leadership	1	1.43
Service leadership	1	1.43
Democratic leadership	1	1.43
Participative leadership	1	1.43
Competent leadership	1	1.43
Emotional intelligence-based leadership	1	1.43
Integral leadership	1	1.43
Resilient leadership	1	1.43
Collaborative leadership	1	1.43
Exemplary adaptive leadership	1	1.43
Adaptive and innovative leadership	1	1.43
Executive leadership	1	1.43
Participative	1	1.43

Source: own elaboration.

**Table 6:** Types of leadership.

Leadership styles	Abs freq.	Rel freq. (%)
Transformational leadership	48	69.57
Transactional and task-oriented leadership	12	17.39
Contextual and adaptive leadership	5	7.25
Participative and democratic leadership	4	5.8

Source: own elaboration.

**Table 7:** Mapping characteristics to transformational leadership dimensions.

Characteristics	Transformational leadership dimension
Care about nurses as individuals	IC
Being a communicator	IC
Empowerment	IC
Sharing decision making	IC
Coaching	IC
Practicing according to a nursing administration standard	IC
Empathy	IC
Providing autonomy	IC
Accessibility	IC
Listening	IC
Emotional intelligence	IC
Building positive relationships with your staff	IC
Cultural competence	IC
Effective conflict management	IC
Establishing core values of the nursing team and teamwork	II
Will and commitment	II
Ethics	II
Self-control	II
Serving as a role model for staff	II
Credibility	II
Trustworthiness	II
Charismatic	II
Visibility	II
Resilience	II
Being visionary	IM
Outcomes-oriented	IM
Inspiring	IM
Enthusiasm	IM
Passion	IM
Motivating	IM
Being a change agent	IM
Knowledgeable	IS
Innovation	IS
Promoting strategies where staff can use ICT	IS
Creativity	IS
Understanding healthcare economics knowledge	IS
Adaptive	IS
Being a critical thinker	IS

Source: own elaboration. Legend: IC: individualized consideration; II: idealized influence; IM: inspirational motivation; IS: intellectual stimulation.

**Table 8:** Impact and applications of leadership in nursing.

Leadership style	Impact on job satisfaction	Efficiency in patient care	Conflict management	Team commitment	Adaptation to change
Transformational leadership	High	Significantly improved	Effective	Significantly improved	High
Transactional and task-oriented leadership	Moderate to high	Maintained or improved with incentives	Effective with clear structure	Moderate to high	Moderate to low
Contextual and adaptive leadership	Variable	Highly adaptable to changing situations	Variable	Highly adaptable	Very high
Participative and democratic leadership	High	Improved with inclusive decision-making	Very effective in collaborative environments	High	High

Source: own elaboration.

## Data Availability

The datasets generated during and/or analyzed during the current study are available from the corresponding author upon request.
